# Long-term immunological responses to treatment among HIV-2 patients in Côte d’Ivoire

**DOI:** 10.1186/s12879-020-4927-x

**Published:** 2020-03-12

**Authors:** Peter A. Minchella, Christiane Adjé-Touré, Guoqing Zhang, Andre Tehe, Judith Hedje, Erin R. Rottinghaus, Natacha Kohemun, Micheline Aka, Karidia Diallo, G. Laissa Ouedraogo, Kevin M. De Cock, John N Nkengasong

**Affiliations:** 1 Division of Global HIV and Tuberculosis, Centers for Disease Control and Prevention, 1600 Clifton Rd., Atlanta, GA USA; 2 Division of Global HIV and Tuberculosis, Centers for Disease Control and Prevention, Abidjan, Côte d’Ivoire; 3 Division of Global HIV and Tuberculosis, Centers for Disease Control and Prevention, Pretoria, South Africa; 4 Division of Global HIV and Tuberculosis, Centers for Disease Control and Prevention, Nairobi, Kenya; 5 Africa Centres for Disease Control and Prevention, Addis Ababa, Ethiopia

**Keywords:** HIV, HIV-2, CD4, ART, Côte d’Ivoire, Africa

## Abstract

**Background:**

Studies indicate that responses to HIV-2 treatment regimens are worse than responses to HIV-1 regimens during the first 12 months of treatment, but longer-term treatment responses are poorly described. We utilized data from Côte d’Ivoire’s RETRO-CI laboratory to examine long-term responses to HIV-2 treatment.

**Methods:**

Adult (≥15 years) patients with baseline CD4 counts < 500 cells/μl that initiated treatment at one of two HIV treatment centers in Abidjan, Côte d’Ivoire between 1998 and 2004 were included in this retrospective cohort study. Patients were stratified by baseline CD4 counts and survival analyses were employed to examine the relationship between HIV type and time to achieving CD4 ≥ 500 cells/μl during follow up.

**Results:**

Among 3487 patients, median follow-up time was 4 years and 57% had documented ART regimens for > 75% of their recorded visits. Kaplan-Meier estimates for achievement of CD4 ≥ 500 cells/μl after 6 years of follow-up for patients in the lower CD4 strata (< 200 cells/μl) were 40% (HIV-1), 31% (HIV-dual), and 17% (HIV-2) (log-rank *p* < 0.001). Cox Regression indicated that HIV-1 was significantly associated with achievement of CD4 ≥ 500 cells/μl during follow-up, compared to HIV-2.

**Conclusions:**

Sub-optimal responses to long-term HIV-2 treatment underscore the need for more research into improved and/or new treatment options for patients with HIV-2. In many West African countries, effective treatment of both HIV-1 and HIV-2 will be essential in the effort to reach epidemic control.

## Background

The vast majority of individuals with HIV-2, including those dually reactive with HIV-1 and HIV-2 (HIV-D), are in West Africa, where the HIV-2 virus was first isolated in 1986 [1]. HIV-2 is generally considered to be less virulent than HIV-1; and HIV-2 infections typically have longer stages of clinical latency [2], slower CD4 depletion rates [3], and lower viral loads [4, 5]. Despite this, many patients with HIV-2 progress to AIDS [6], acquire opportunistic infections [7], and experience AIDS-related mortality.

Historically, antiretroviral therapy (ART) guidelines for patients with HIV-2 have been based on ‘low quality’ evidence, such as observational studies, case reports, and in vitro studies [8, 9]. Broadly, this evidence has shown that HIV-2 is resistant to non-nucleoside reverse transcriptase inhibitors (NNRTIs) [10], shows decreased susceptibility to some protease inhibitors (PIs) [11, 12], and is susceptible to a variety of nucleoside reverse transcriptase inhibitors (NRTIs), boosted PIs, and integrase inhibitors [13]. Recent data indicate that the integrase strand transfer inhibitor (INSTI) class of integrase inhibitors are safe and effective against HIV-2 [14, 15]. While reports suggest that boosted PI-containing regimens perform better that triple NRTI regimens [16], it has been challenging to identify a preferred initial ART regimen for patients with HIV-2 [17] – which is reflected by comparatively poor responses to treatment among those patients. Specifically, reports indicate that both reduction in viral load [18] and CD4 recovery [19] are sub-optimal for HIV-2 patients during the first 12 months of ART, compared to patients with HIV-1. Diminished CD4 recovery in patients with HIV-2 was even observed after adjustment for pretreatment plasma viral load [20], which some hypotheses credit as the cause of poor CD4 recovery.

While this evidence makes a strong case for development of improved treatment options for patients with HIV-2, there is a clear gap in the data when it comes to long-term responses to treatment. Only a few HIV-2 studies exceed 12 months of follow-up [21–23], limiting understanding of how responses to treatment evolve over time. We aimed to address this gap via a retrospective cohort study that assessed long-term CD4 recovery according to HIV type in Côte d’Ivoire, a West African country where 5% of HIV-infected adults are estimated to have HIV-2 or HIV-D infection [24].

## Methods

### Setting

Starting in 1998, with support from the UNAIDS Drug Access Initiative (DAI), the Côte d’Ivoire Ministry of Health implemented a pilot ART program that was designed to serve as a model for providing ART in low-resource settings. Seven treatment centers in Abidjan, Côte d’Ivoire were accredited to provide ART to patients that met sociodemographic and biological eligibility criteria and all laboratory testing for specimens referred from those patients was conducted at the RETRO-CI laboratory, also in Abidjan [25].

Project RETRO-CI is a collaboration between the U.S Centers for Disease Control and Prevention (CDC) and the Ivorian Ministry of Health that provides laboratory testing and data management support to the clinics referring patient specimens to the RETRO-CI laboratory.

### Study design and population

Adult (≥15 years) patients with documented HIV types and baseline CD4 counts < 500 cells/μl that initiated ART at one of two HIV treatment centers between 1998 and 2004 were included in this retrospective cohort study. The treatment centers, Unité de Soins Ambulatoire et de Conseil (USAC) and Service de Maladies Infectieuses et Tropicales, were located at Treichville University Hospital (UTH) in Abidjan and accredited to provide ART as part of the initial DAI pilot. The remaining five treatment centers that were accredited as part of the DAI pilot were not included in this study. The baseline CD4 count was defined as that recorded as part of the *bilan initial* or ‘initial visit’ for each patient in the RETRO-CI database. Data on patient demographics and laboratory testing results were extracted from the RETRO-CI laboratory database. Data on treatment regimens were also extracted from the database, though only the presence/absence of initial treatment regimens were used, as details of specific regimens were not recorded with the intention of serving as a primary data source.

### Outcomes and exposure variables

The primary outcome was achievement of CD4 ≥ 500 cells/μl, which has been linked to a reduced risk of clinical progression for patients on ART [26]. Patients were categorized by HIV type and stratified by baseline CD4 counts. Patient demographics, baseline CD4 counts, and the ART clinic at which the patient initiated treatment were factors included in multivariable models.

### Data analysis

Data were analyzed using SAS 9.4 (SAS Institute Inc., Cary, NC). The dataset utilized may be requested from the RETRO-CI laboratory and the Ivorian Ministry of Health.

Frequencies were generated for categorical variables and means, standard deviations, medians, and interquartile ranges (IQR) for normally distributed and non-normally distributed continuous variables, respectively.

Survival analyses were employed to examine the relationship between HIV type and time to achieving CD4 ≥ 500 cells/μl during follow up in two baseline CD4 strata. Patients were censored if the study period closed prior to achievment of CD4 ≥ 500 cells/μl. The end of the study period was July 31, 2015. Kaplan-Meier (KM) methods were used to estimate and visualize survival probabilities as a function of time and log-rank tests were utilized to compare KM curves across HIV types. Cox proportional hazards regression models were used to estimate crude and adjusted hazard ratios (HR and aHR) and 95% confidence intervals (CI) for exposure variables. HIV-2 served as the reference category for all models.

## Results

Demographics and characteristics for 3487 patients included in this study are summarized in Table 1. Overall, compared to their counterparts in the lower CD4 strata (< 200 cells/μl), patients in the upper CD4 strata (200–500 cells/μl) had longer median follow-up times, were younger, and were more likely to be female. Within-CD4 strata comparisons by HIV type revealed that HIV-2 and HIV-D patients were older and more likely to be male.

**Table 1:** Demographics and characteristics of study patients by baseline CD4 strata and HIV type

	Baseline CD4 < 200 cells/μl				Baseline CD4 200–500 cells/μl				
	HIV-1	HIV-2	HIV-D	Total	HIV-1	HIV-2	HIV-D	Total	
Total patients, n	2165	77	108	2350	1049	31	57	1137	3487
Visits, median (IQR)	7.0 (3–19)	7.0 (3–16)	7.0 (3–17)	7.0 (3–19)	8.0 (3–18)	11.0 (3–21)	7.5 (3–18.5)	8.0 (3–18)	7.0 (3–19)
Follow-up time (years), median (IQR)	3.8 (0.7–11.2)	3.0 (0.8–8.0)	2.5 (0.7–10.7)	3.7 (0.7–11.2)	4.5 (1.0–11.3)	5.6 (2.6–10.6)	4.7 (1.4–11.0)	4.6 (1.0–11.3)	4.0 (0.8–11.2)
Year of baseline CD4, n (%)									
1998–2000	444 (20.5)	15 (19.5)	29 (26.9)	488 (20.8)	254 (24.2)	8 (25.8)	11 (19.3)	273 (24.0)	761 (21.8)
2001–2002	768 (35.5)	31 (40.3)	40 (37.0)	839 (35.7)	394 (37.6)	10 (32.3)	27 (47.4)	431 (37.9)	1270 (36.4)
2003–2004	953 (44.0)	31 (40.3)	39 (36.1)	1023 (43.5)	401 (38.2)	13 (41.9)	19 (33.3)	433 (38.1)	1456 (41.8)
CD4 count (cells/μl), median (IQR)	69 (21–127)	69 (42–112)	71 (23–125)	69 (22–126)	299 (246–381)	282 (236–373)	312 (250–382)	299 (246–381)	128 (41–244)
Age (years)									
Age, mean ± SD	37.8 ± 8.7	43.8 ± 8.3	41.4 ± 6.6	38.2 ± 8.7	36.7 ± 9.4	41.3 ± 9.3	39.7 ± 8.3	37.0 ± 9.4	37.8 ± 9.0
< 35, n (%)	837 (38.7)	8 (10.4)	15 (13.9)	860 (36.6)	487 (46.4)	6 (19.4)	17 (29.8)	510 (44.9)	1370 (39.3)
35–44, n (%)	844 (39.0)	39 (50.6)	57 (52.8)	940 (40.0)	358 (34.1)	11 (35.5)	28 (49.1)	397 (34.9)	1337 (38.3)
≥ 45, n (%)	484 (22.4)	30 (39.0)	36 (33.3)	550 (23.4)	204 (19.4)	14 (45.2)	12 (21.1)	230 (20.2)	780 (22.4)
Sex, n (%)									
Female	1107 (51.1)	33 (42.9)	37 (34.3)	1177 (50.1)	626 (59.7)	11 (35.5)	29 (50.9)	666 (58.6)	1843 (52.9)
Male	1058 (48.9)	44 (57.1)	71 (65.7)	1173 (49.9)	423 (40.3)	20 (64.5)	28 (49.1)	471 (41.4)	1644 (47.2)
Percentage of visits with documented ART regimen, n (%)									
< 50%	216 (10.0)	8 (10.4)	12 (11.1)	236 (10.0)	307 (29.2)	8 (25.8)	15 (26.3)	330 (29.0)	566 (16.2)
50–75%	614 (28.3)	26 (33.7)	31 (28.7)	671 (28.6)	229 (21.8)	6 (19.4)	17 (29.8)	252 (22.2)	923 (26.5)
> 75%	1335 (61.7)	43 (55.8)	65 (60.1)	1443 (61.4)	513 (48.9)	17 (54.8)	25 (43.8)	555 (48.8)	1998 (57.3)

Values are reported as median (IQR), mean ± SD, or n(%). Visits and follow-up time refer only to visits recorded in the RETRO-CI laboratory database – if visits did not include specimen referrals to RETRO-CI they were not included. The percentage of visits with a documented ART regimen refers to visits in the RETRO-CI database (not including the initial visit) for which a current ART regimen was recorded

*Abbreviations*: *IQR* Interquartile range, *SD* Standard deviation, *ART* Antiretroviral therapy

### Treatment

Overall, nearly 80% of patients had documented ART regimens at the time of their initial visits (Table 2) and 57% of patients had documented ART regimens for > 75% of their recorded visits (Table 1).

**Table 2:** HIV type and association with achieving CD4 ≥ 500 cells/μl during follow-up

Baseline CD4 count	HIV type	Hazard Ratio (HR)	95% CI	Adjusted HR	95% CI
< 200 cells/ μl	HIV-1	3.36	1.68–6.75	2.60	1.29–5.22
	HIV-D	2.11	0.94–4.74	2.22	0.99–4.99
	HIV-2	–	–	–	–
200–500 cells/μl	HIV-1	1.64	0.92–2.91	1.42	0.79–2.55
	HIV-D	1.47	0.74–2.92	1.32	0.65–2.66
	HIV-2	–	–	–	–

Univariate and multiple variable logistic regression were utilized to model the relationship between HIV-type and achievement of CD4 ≥ 500 cells/μl during follow-up. The adjusted model includes age, sex, baseline CD4 count, year of baseline CD4 count, and HIV clinic as covariates

### Achievement of CD4 ≥ 500 cells/μl

**Fig. 1:**
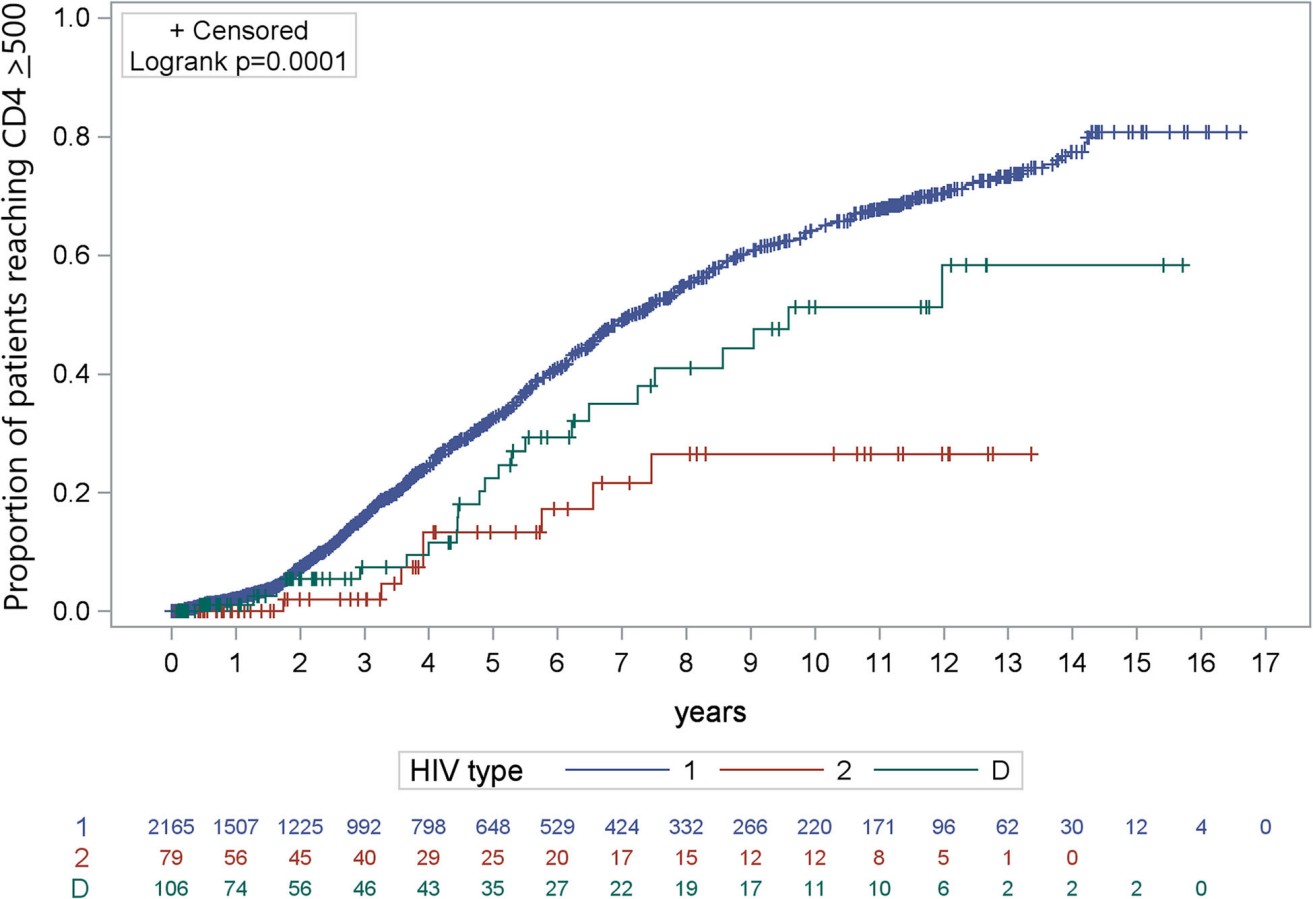
Time to achieving CD4 ≥ 500 cells/μl among patients with baseline CD4 < 200 cells/μl by HIV type

**Fig. 2:**
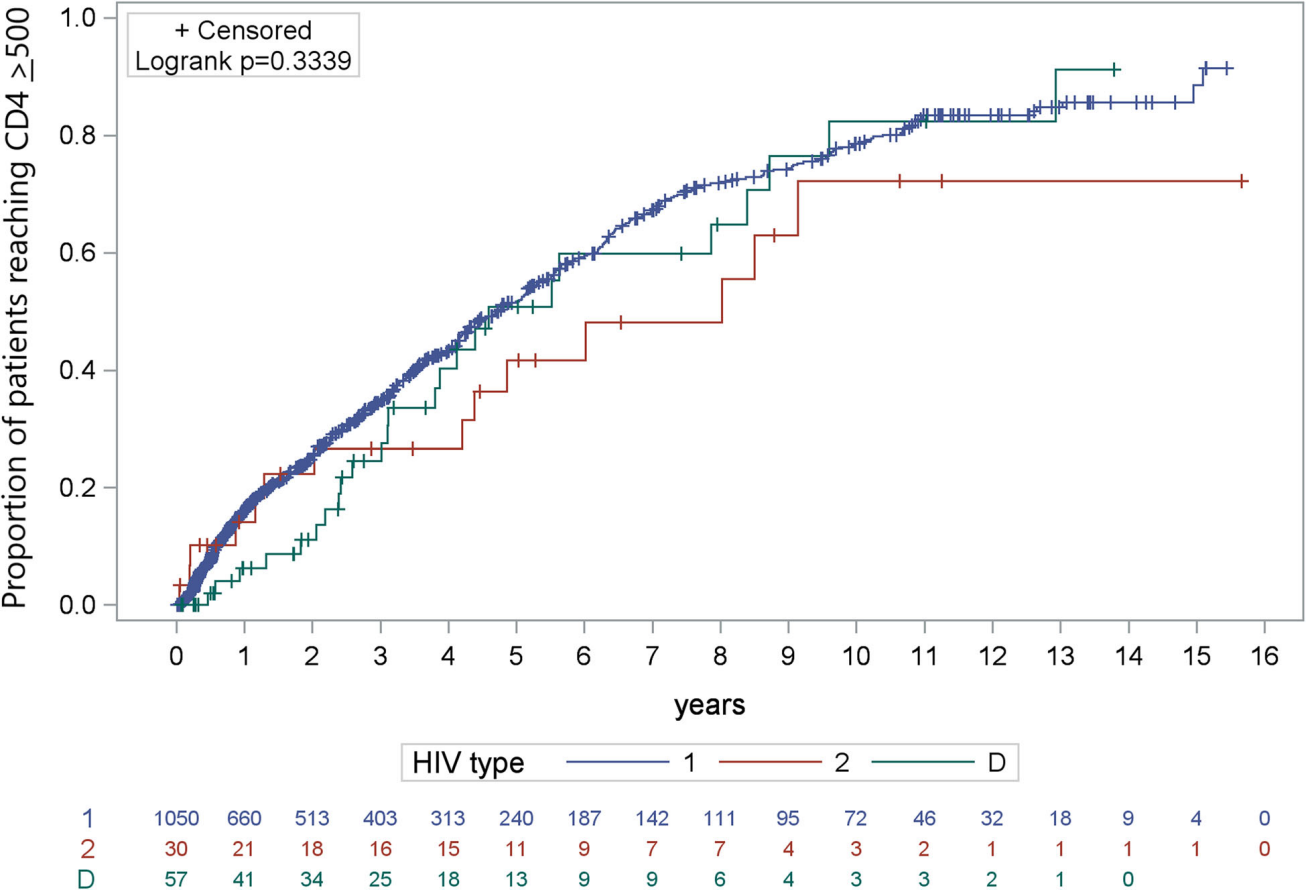
Time to achieving CD4 ≥ 500 cells/μl among patients with baseline CD4 200–500 cells/μl by HIV type

For patients in the lower CD4 strata, KM estimates for achievement of CD4 ≥ 500 cells/μl after 3 and 6 years were highest amongst those with HIV-1 (3 years = 15%; 6 years = 40%), followed by HIV-D (3 years = 6%; 6 year = 31%) and HIV-2 (3 years = 2%; 6 years = 17%) (log-rank *p* < 0.001; Fig. 1). KM estimates for achievement of CD4 ≥ 500 cells/μl in the upper CD4 strata followed a similar trend (HIV-1, 3 years = 30%, 6 years = 57%; HIV-D: 3 years = 23%, 6 years = 54%; HIV-2: 3 years = 20%, 6 years = 42%), though the KM curves were not significantly different (log-rank *p* = 0.33; Fig. 2).

### Factors associated with achievement of CD4 ≥ 500 cells/μl

Among 2350 patients in the lower CD4 strata HIV-1 was significantly associated with achieving CD4 ≥ 500 cells/μl during follow-up compared to HIV-2 (Table 2). HIV-D was also positively associated with achieving CD4 ≥ 500 cells/μl during follow-up compared to HIV-2, but the associations were not significant. There was not a significant association between HIV type and achieving CD4 ≥ 500 cells/μl during follow-up in the upper CD4 strata.

## Discussion

Among a well-characterized cohort of HIV-infected patients with low baseline CD4 counts in Côte d’Ivoire, those with HIV-1 were more likely than those with HIV-2 to achieve CD4 ≥ 500 cells/μl during follow-up. These results reinforce the importance of early ART initiation for all HIV types and underscore the need for improved HIV-2 treatment options and HIV-2 treatment guidelines.

Historically, low prevalence and geographic restrictions limited HIV-2 studies to small sample sizes and short follow-up periods. This study was unique in that it included long follow-up periods for relatively large numbers of HIV-2 patients, demonstrating the utility of databases such as the one maintained at the RETRO-CI laboratory and providing an opportunity to assess responses to treatment over time. In that context, we found that immunological responses among patients with HIV-2 were sub-optimal in a cohort of patients with low baseline CD4 counts and a median follow-up period of nearly 4 years.

While the mechanisms responsible for sub-optimal treatment responses are beyond the scope of this study, our results hint at some possible scenarios. The most notable are related to CD4 recovery plateaus and the relationship between baseline and peak CD4 counts, both of which have been described in responses to treatment among HIV-1 patients with suppressed viral loads [27–29]. Specifically, since HIV-2 patients tend to present with lower viral loads [4, 5] following longer periods of clinical latency [2], CD4 recovery plateaus may be lower and/or already achieved by the time they initiate treatment, potentially contributing to the sub-optimal treatment responses that we observed. Our results for patients in the upper CD4 strata, in which HIV-type did not predict achievement of CD4 ≥ 500 cells/μl during follow-up, are in line with this general hypothesis.

The findings in this study highlight the need for improved ART options for patients with HIV-2, a need which the HIV community is beginning to address. Recent publication of results from two trials of integrase inhibitor-based ART for HIV-2 [30, 31] suggest that both the quality of evidence and the efficacy of ART regimens will improve over time. However, HIV-2’s geographic restriction to mostly low-resource settings in West Africa means that improved evidence, better treatment options, and updated guidelines are only part of the challenge of improving responses to HIV-2 treatment. Implementation of new treatment options in these settings will also need to include efforts to ensure that recommended ART options are available, prescribed correctly, and monitored over time.

Key strengths of this study included the large, well-characterized cohort and the length of follow-up. These data contribute to a better understanding of long-term responses to treatment among HIV-2 patients, support early ART initiation for all HIV types, and highlight the need for improved treatment options and guidelines for patinets with HIV-2. Limitations included the lack of reliable data describing patient treatment regimens and the lack of data characterizing responses to treatment, including routine viral load results and descriptions of patient outcomes. Limited availability of HIV-D patient samples for retesting was also a limitation, as studies suggest that it can be challenging to differentiate HIV-D patients from patients with HIV-1 (or HIV-2) mono-infection [32, 33]. The composition of the RETRO-CI database, which was maintained as part of routine laboratory testing, was also a limitation. It did not include variables that may have provided context to the data (such as laboratory methods). Finally, the study period was a limitation, as the early part of this period was one during which clinicians in Côte d’Ivoire prescribed non-recommended ART regimens in hopes of improving responses to treatment. This has been noted in prior studies in Côte d’Ivoire [34] and implicated as a cause of poor responses to treatment among HIV-2 patients [35].

## Conclusions

HIV-1 patients with low baseline CD4 counts in Côte d’Ivoire were more likely to achieve CD4 recovery during long-term follow-up than those with HIV-2. These findings likely reflect limited efforts to develop, optimize, and perscribe ART regimens specifically for HIV-2 and underscore the need for improved options. While the burden of HIV-2 is relatively small, it nevertheless contributes to HIV epidemics in many West African countries and must be treated effectively alongside HIV-1 in order for those countries to reach epidemic control.

## Data Availability

Data are owned by the Côte d’Ivoire Ministry of Health and may be requested by qualified researchers.

## Abbreviations

ART: Antiretroviral therapy
CDC: Centers for Disease Control and Prevention
CI: Confidence intervals
DAI: UNAIDS Drug Access Initiative
HIV-D: Dually reactive with HIV-1 and HIV-2
INSTI: Integrase strand transfer inhibitor
IQR: Interquartile ranges
KM: Kaplan-Meier
NNRTI: Non-nucleoside reverse transcriptase inhibitor
NRTI: Nucleoside reverse transcriptase inhibitor
PI: Protease inhibitor
USAC: Unité de Soins Ambulatoire et de Conseil
UTH: Treichville University Hospital
